# Understanding growth and age of red tree corals (*Primnoa pacifica*) in the North Pacific Ocean

**DOI:** 10.1371/journal.pone.0241692

**Published:** 2020-12-01

**Authors:** Emma Choy, Kelly Watanabe, Branwen Williams, Robert Stone, Peter Etnoyer, Ellen Druffel, Thomas Lorenson, Mary Knaak

**Affiliations:** 1 W.M. Keck Science Department of Claremont McKenna, Pitzer, and Scripps Colleges, Claremont, CA, United States of America; 2 Alaska Fisheries Science Center, National Marine Fisheries Service, NOAA, Juneau, AK, United States of America; 3 NOAA National Centers for Coastal Ocean Science, Charleston, SC, United States of America; 4 Department of Earth System Science, University of California Irvine, Irvine, CA, United States of America; 5 USGS Pacific Coastal and Marine Science Center, Santa Cruz, CA, United States of America; University of Bologna, ITALY

## Abstract

Massive, long-lived deep-sea red tree corals (*Primnoa pacifica*) form a solid, layered axis comprised of calcite and gorgonin skeleton. They are abundant on the outer continental shelf and upper slope of the Northeast Pacific, providing habitat for fish and invertebrates. Yet, their large size and arborescent morphology makes them susceptible to disturbance from fishing activities. A better understanding of their growth patterns will facilitate *in-situ* estimates of population age structure and biomass. Here, we evaluated relationships between ages, growth rates, gross morphological characteristics, and banding patterns in 11 colonies collected from depths of ~141–335 m off the Alaskan coast. These corals ranged in age from 12 to 80 years old. They grew faster radially (0.33–0.74 mm year^-1^) and axially (2.41–6.39 cm year^-1^) than in previously measured older colonies, suggesting that growth in *P*. *pacifica* declines slowly with age, and that basal diameter and axial height eventually plateau. However, since coral morphology correlated with age in younger colonies (< century), we developed an *in-situ* age estimation technique for corals from the Northeast Pacific Ocean providing a non-invasive method for evaluating coral age without removing colonies from the population. Furthermore, we determined that annual bands provided the most accurate means for determining coral age in live-collected corals, relative to radiometric dating. Taken together, this work provides insight into *P*. *pacifica* growth patterns to inform coastal managers about the demographics of this ecologically important species. With this new ability to estimate the age of red tree corals *in-situ*, we can readily determine the age-class structure and consequently, the maturity status of thickets, using non-invasive video survey techniques when coupled with mensuration systems such as lasers or stereo-cameras. Enhanced surveys could identify which populations are most vulnerable to disturbance from human activities, and which should be highlighted for protection.

## Introduction

Fishing practices, including bottom trawling and long lining, can disturb benthic ecosystems, particularly those where the seafloor is highly structured with large sedentary invertebrates such as corals and sponges [1–4]. Gorgonian corals are especially vulnerable to fishing practices due to easy ensnarement of this large arborescent sea-fan, which adheres to the seafloor with a single holdfast. Some gorgonians are very long-lived and communities of older colonies compromised by fishing gear may take decades to centuries to recover [5–9]. Understanding how fast these corals grow and the relationship between size and age can provide estimates of recovery times of these communities [2, 10, 11]. This is critically important in areas vulnerable to fishing disturbance such as those in the Northeast Pacific Ocean where the importance of these habitats to fisheries has been documented [3, 4, 12].

Gorgonian coral communities are important components of the sea floor because they provide habitat for a diversity of invertebrates and fishes [13, 14]. In fact, these corals can serve as habitat engineers [15]: in their absence, shallow-water assemblages shift from predominately corals and sponges to algae and turf-forming species [16]. Furthermore, some gorgonian corals are indicator species, and in addition to disturbance from fishing activities are sensitive to warming ocean temperatures [16, 17] and oil pollution [18, 19]. The conservation of these corals is thus critical for maintaining ecological diversity and community resilience.

The gorgonian *Primnoa pacifica* [20], also known as the red tree coral, are ecologically important deep-sea corals in the North Pacific Ocean [4]. They have been referred to as “keystone species,” “foundation species,” and “ecosystem engineers” [4, 21]. These animals are comprised of an internal skeleton arising from the holdfast attached directly to hard substrate. Their skeleton is largely comprised of protein-rich organic gorgonin sometimes interspersed with calcite. The source of elements to the gorgonin skeleton is organic material produced in surface waters and transported to depth to be fed upon by the corals. In contrast, the calcite elements are sourced from ambient seawater at depth [22–24]. A thin layer of coenenchyme with polyps covers the entirety of the skeleton. The skeletal central axis grows axially and radially, such that through time the coral grows taller along its axial axis and adds layers to the outside of its skeletal trunk increasing the trunk diameter. They can grow to massive size (greater than 2 m in height [25]), in part because of their long lifespans that can exceed a century or more [26].

In the central skeletal axis of some gorgonian corals, concentric couplets of gorgonin-calcite bands form annually, providing a means to determine the age of a colony; however, fine-scale bands of unknown periodicity are also present, indicating possible drivers of skeletal banding [9, 26–29]. The finer bands may reflect variations in the color of the organic skeleton (which in turn reflects the degree of protein cross-linkages during skeletal formation) and/or alternations of the gorgonin with calcite skeleton [9, 30, 31]. In addition to annual growth band counts, radiometric dating (^14^C and ^210^Pb) can provide estimates of coral age, albeit with potential uncertainties depending on the age and collection date of the coral [24, 26, 32, 33].

Previous studies using a combination of annual growth band counts and radiometric dating in *P*. *pacifica* yielded different estimates of radial growth rates ranging from 0.14 to 0.57 mm yr^-1^ [26, 34, 35]. Axial growth rates for this species have only been reported for two specimens, and those estimates ranged from 1.60 to 2.32 cm yr^-1^ [26]. Obtaining axial growth rates in a much larger sample of corals is key to determine the rate of recovery of disturbed communities.

Therefore, the objectives of this study were to 1) determine the age and growth (radial and axial) for a suite of colonies collected in the Northeast Pacific Ocean with morphological data; 2) develop and evaluate age estimate calculations converting morphological data into age; 3) evaluate annual growth band counts and radiometric dating as age determination techniques; and 4) examine the periodicity and potential drivers of sub-annual banding in the axial skeleton. As a whole, this work provides critical insight into recovery times of *P*. *pacifica* which helps inform management policies of important deep-sea coral habitats.

## Materials and methods

### Sample sites and collection

**Fig 1:**
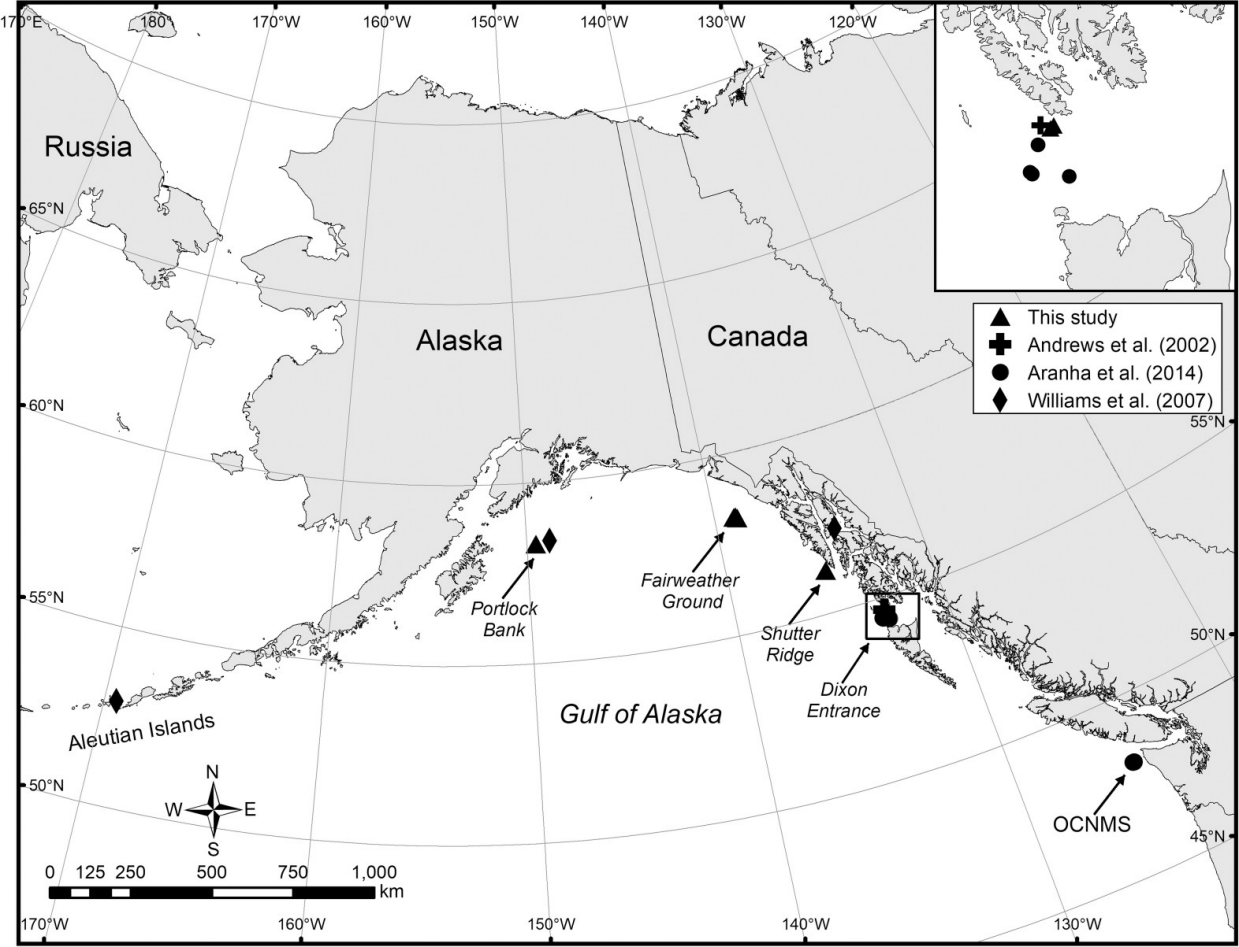
Map showing the collection sites of *Primnoa pacifica* from the current project relative to previous studies (Andrews et al., 2002 [26]; Aranha et al., 2014 [34]; Williams et al., 2007 [35]). Map created by Michele Masuda.

**Fig 2:**
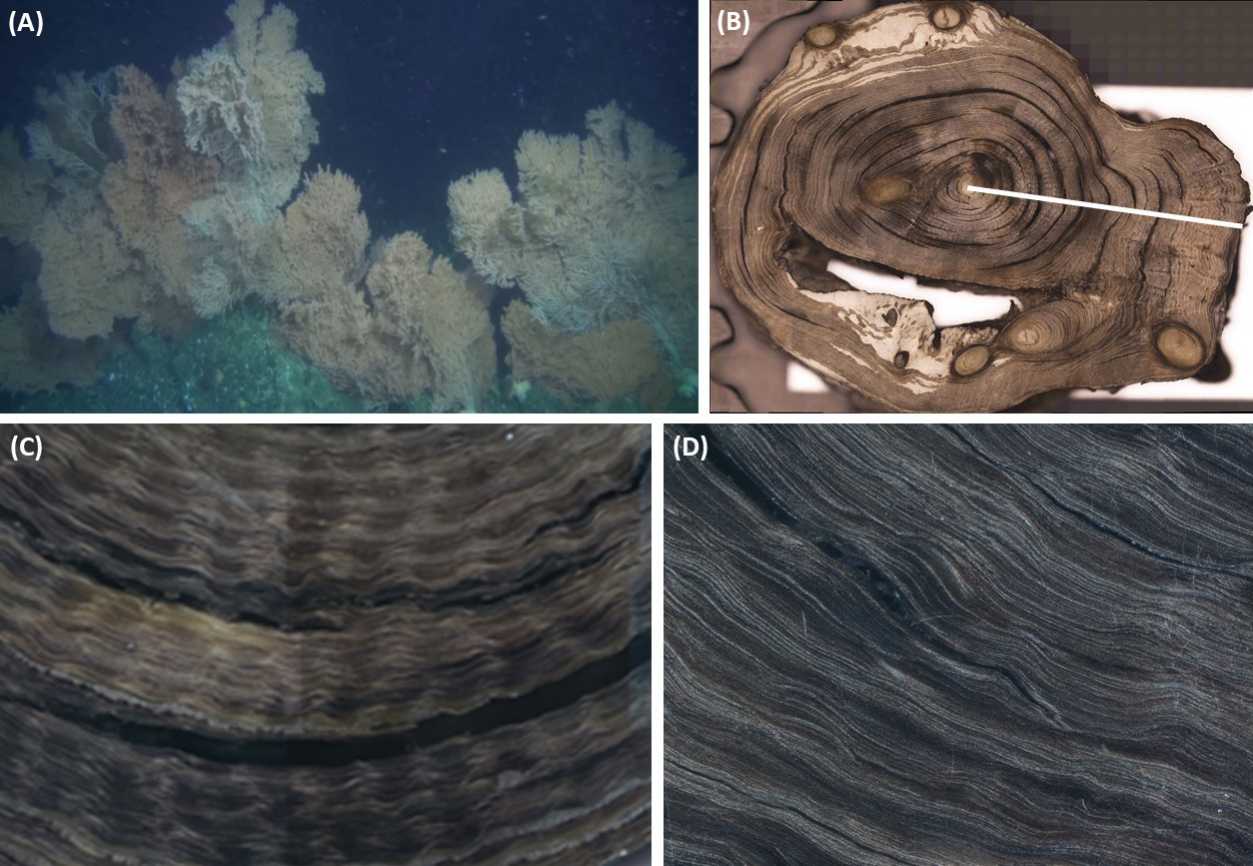
(A) *Primnoa pacifica* colonies on the Shutter Ridge at a depth of 180 m, (B) sub-section from specimen WPA 005 with a white line showing region of axis used to count growth bands, (C) image showing annual growth bands and (D) sub-annual growth bands.

Three intact colonies were collected in 2013 using the remotely operated vehicle (ROV) H2000 deployed from the F/V *Alaska Provider* (Table 1; Figs 1 and 2). Seven intact colonies were collected in 2015 using the ROV Zeus II deployed from the R/V *Dorado Discovery* (Table 1, Fig 1). Additionally, a single specimen (GOA 004; Table 1) was collected with a research bottom trawl deployed from the F/V *Alaska Provider* just prior to the 2013 cruise. Fieldwork research was performed by NOAA’s Alaska Fisheries Science Center and Deep Sea Coral Research and Technology Program under the authority of the U.S. Department of Commerce. Colonies were air dried on board the vessels and morphological data were recorded, including maximum height, basal diameter, wet weight, and distance from the base to first branch.

**Table 1:** Sample ID, collection information, and morphological data for the 11 coral colonies included in this study. Age estimates for all specimens are derived from annual growth bands. Dates are mm/dd/yyyy.

Sample ID	Collection date	Locality	Latitude	Longitude	Depth (m)	Height (cm)	Width (cm)	BaseD (mm)	DistToBr* (cm)	Wet weight (kg)	Age estimate (years)
WPA 001	6/4/2015	Fairweather Grand	58.2457	-138.9045	141	123	67	32.5	7	2.24	26 ± 2
WPA 002	6/5/2015	Fairweather Grand	58.2458	-138.9044	142	171	60	25.5	62	1.25	28 ± 2
WPA 003	6/5/2015	Fairweather Grand	58.2377	-138.9894	147	110	70	45.0	30	n/a	19 ± 2
WPA 004	6/7/2015	Fairweather Grand	58.2047	-138.8175	163	223	101	53.0	29.5	20.45	80 ± 1
WPB 005	6/9/2015	Dixon Entrance	54.6252	-132.8828	335	160	98	49.0	29	16.6	67 ± 4
WPB 006	6/8/2015	Dixon Entrance	54.6345	-132.8510	164	99	26	19.0	20	1.14	16 ± 2
WPB 007	6/8/2015	Dixon Entrance	54.6345	-132.8510	164	87	23	15.0	17	0.64	15 ± 1
GOA 004	7/19/2013	Portlock Bank	58.3102	-149.5087	147	78	50	15.0	16	1.08	14 ± 2
GOA 011	8/13/2013	Shutter Ridge	56.1749	-135.1165	191	65	24	10.0	8.5	0.62	12 ± 2
GOA 022	8/13/2013	Shutter Ridge	56.1722	-135.1160	203	72	32	16.0	12.3	1.13	18 ± 1
GOA 067	8/15/2013	Shutter Ridge	56.1784	-135.1180	214	153	121	45.0	32	n/a	31 ± 2

*Distance to first branch.

### Sample preparation

In the laboratory, a diamond-edge saw was used to cut three adjacent 0.25–0.50 cm cross-section discs from the basal portion (holdfast) of each coral. One cross-section from each coral was mounted on a glass slide and polished for annual band counts and digital imaging, one cross-section was used for dissected band counts and radiocarbon dating, and one cross-section was used for ^210^Pb dating. Although growth rings were only counted in two of the three cross sections, it is assumed that all cross sections contain the same number of growth bands since they were cut from adjacent parts of the coral. The two cross-sections used for (1) dissected band counts and radiocarbon dating and (2) ^210^Pb dating were bathed in 100 ml of 5% HCl solution for a minimum of 10 days (up to four weeks). The HCl solution was refreshed every other week for four weeks so that all the calcite bands layered between the organic skeleton had dissolved. After acidification, the sample was transferred to soak in Milli’Q water for band peeling.

### Photographed band counts

Using the mounted and polished cross-sections (Fig 2), the number of annual bands along the longest radial transect were counted by two researchers under a light microscope for all specimens in this study. The radial growth rate (mm year^-1^) of each coral was obtained by dividing the base diameter of the coral by the age of the coral, as determined by annual growth bands. Similarly, axial growth rates (cm year^-1^) were calculated by dividing the maximum height of the coral by the age of the coral, as determined by annual growth bands.

The cross sections were then imaged using a Nikon digital microscope with NIS-Elements, a Nikon microscope software package. Using these high-resolution images, the total number of sub-annual bands were counted along the same longest radial transect for four specimens (GOA 011, WPA 002, WPA 004, and WPB 005) by two researchers (S1 File). Four larger specimens were chosen to encompass a range in sizes and the ability to cut adjacent cross sections. The bands were sometimes difficult to distinguish; when major discrepancies in band counts were evident, researchers re-counted bands collaboratively, discussing the presence or absence of a band when uncertainties arose.

### Dissected band counts

Using one of the HCl-bathed cross-sections, sub-annual growth bands were peeled and counted in sections from the outside to the center of the coral cross-section using forceps and working under a microscope at 30x magnification. Without tearing the bands, sections were peeled with the least number of bands possible, with each section containing approximately 1–20 bands depending on the ease of band separation. If possible, sections were peeled all the way around the circumference of the sample. The separated sections were air dried and packaged into labeled weigh paper packets.

### Radiocarbon analysis

For radiocarbon sample preparation, a laminar flow hood workspace was cleaned with deionized water. All glassware was soaked in 10% HCl for a minimum of 1 hour. The glass vials used for radiocarbon dating were additionally combusted at 540 ˚C for 2 hours, while the vial caps were washed with soap and water, and then acid washed in 10% HCl for 30 seconds and rinsed with water. After sub-annual bands were dissected and counted, three of the four colonies (WPA 002, WPB 005, and WPA 004) were selected for radiocarbon analysis. Based on annual band counts, colony GOA 011 was too young for radiocarbon dating to be effective. Five milligram sub-samples reflecting no more than 10% of the entire sample and equidistant from each other based on dissected band count numbers were pulverized into acid washed glass vials. The outermost bands of each sample’s cross-section were analyzed to determine the Δ^14^C values at the time of collection. The ^14^C was measured at the Keck Carbon Cycle Accelerator Mass Spectrometry at the University of California, Irvine. Five milligrams of a National Institute of Standards and Technology (NIST) wood standard (Firi H) and a coal standard were also prepared as a reference for radiocarbon analysis and sample preparation backgrounds were subtracted based on these measurements. All results were corrected for isotopic fractionation according to the conventions of Stuiver and Polach (1977) [36]. Δ^14^C values were assigned a year using the following equation:

$$Year=\frac{\left(\Delta^{14}C-{\Delta C}_{\mathrm{lo}\;A}\right)\left(Year_{\mathrm{hi}\;A}-Year_{\mathrm{lo}\;A}\right)}{{\Delta C}_{\mathrm{hi}\;A}-\Delta^{14}C_{\mathrm{lo}\;A}}+Year_{\mathrm{lo}\;A}$$ (1)

where *Year* is the associated year calculated for this study, Δ^14^C is the measured value of the coral sub-section being dated, Δ^14^C_lo A_ and *Year*_lo A_ are the lower estimates of Δ^14^C and calculated year, and Δ^14^C_hi A_ and *Year*_hi A_ are the upper estimates of Δ^14^C and calculated year (data from Andrews et al., 2013 [37]). Radiocarbon records from Andrews et al. (2013) [37] were constructed using Δ^14^C data from otoliths of Northeast Pacific yelloweye rockfish (*Sebastes ruberrimus*), Pacific halibut, and known-age abalone shell samples. A loess curve, including 95% confidence intervals, was fit to the Δ^14^C otolith data of Northeast Pacific yelloweye rockfish to produce a radiocarbon bomb curve (S1 Fig in S1 File). The yelloweye rockfish are bottom dwelling fish that were collected in waters off southeast Alaska [38]. The rockfish chronology identified the initial rise in ^14^C in the late 1950s and peak values in the late 1960s/early 1970s, which agrees with the ^14^C reconstruction by Roark et al. (2005) [23] for a bamboo coral from the northwestern coast of Canada.

### Lead-210 dating

Measurements of ^210^Pb activity provided a third method for evaluating coral age for specimens WPA 002, WPA 004, and WPB 005. Specimens were rinsed thoroughly in Milli-Q water and then dried on a clean watch glass in a laminar flow hood. While still malleable enough to be manipulated, the specimens were split into three sections for sub-sampling (exterior, middle, and interior). Subsamples were dried, cooled in liquid nitrogen, and pulverized in a Genogrinder. Subsamples were weighed (~ 1 g) and placed in clean, new 4-ml vials. Due to the smaller size of the cross-sections initially cut, it was necessary to cut a second cross-section for WPA 002 and WPA 004, such that the sub-samples for these specimens were a combination of two cross-sections. All prepared ^210^Pb samples were sent to the United States Geological Survey at the Pacific Coastal and Marine Science Center for ^210^Pb dating using a germanium gamma ray detector. ^210^Pb decays at a constant rate yet is also in secular equilibrium with ^226^Ra. To determine the specimen age from ^210^Pb values, the excess amount of ^210^Pb (^210^Pb_ex_), ultimately derived from deposition of atmospheric ^222^Rn, which in turn decays to ^210^Pb, is determined by subtracting measured ^226^Ra activity. ^210^Pb_ex_ decays according to the law of radioactive decay:

$$A_{ex}=A_{o}e^{-\lambda t}$$ (2)

where *A_ex_* is the measured ^210^Pb_ex_ at time t, *A_o_* is the initial ^210^Pb_ex_ at time 0, and *λ* is 0.0311 or the natural log of 2 divided by the half-life of ^210^Pb (22.3 years). Inputting the calculated ^210^Pb_ex_ values for each specimen into the equation above yields the age of each sample.

### Data analysis

The relationship between specimen age derived from annual band counts and the morphological data was quantified by comparing linear and logarithmic fits to find the best fit model using R software. For height, width, and basal diameters, these equations represent a means to convert a measurement of a morphological parameter into specimen age. We primarily focus on basal diameter because this metric has most often been reported in corals that also have their ages determined. We validated these age estimation calculations in three ways: 1) We applied the calculations to ten previously collected and aged corals with available radii data that were less than a century old from the northeast Pacific Ocean ([34, 35]; Table 3). Basal diameter was determined from the radius in these corals (assumed diameter = 2*radius, although see discussion on this assumption below). 2) We developed and validated a second calculation relating basal diameter to age using all available colonies (< century in age) for the northeast Pacific Ocean. 3) We apply this regional age estimation to corals collected from the western Pacific Ocean [39]. For each of these comparisons, we evaluated how well the ages derived from the basal estimates correspond to the ages derived from annual band counts or ^210^Pb dating.

In the three specimens with 14C-dating (WPA 002, WPA 004, WPB 005), we calculated banding frequencies and sub-annual radial and axial growth rates. Banding frequencies (growth bands year^-1^) were calculated through time for each colony according to the following equation:

$$Banding\;frequency=\frac{Band\;count_{n}-Band\;count_{n+1}}{Age_{n}-Age_{n+1}},\;n=1,2,3\ldots$$ (3)

Where *Band count* is the average associated growth band count of the sub-section used for radiocarbon dating, *Age*_*n*_ is the radiocarbon age of the older *n* sub-section of the coral, and *n* is the sub-section of the coral sent in for radiocarbon dating (a smaller *n* is closer to the center of the coral representing older skeleton).

## Results

### Morphological data

Eleven intact specimens were collected during two research cruises in 2013 and 2015. Coral height (axial length) ranged from 65 to 223 cm, width ranged from 23 to 121 cm, and basal diameter ranged from 1 to 5.3 cm (Table 1). Many of the morphological parameters significantly correlated with each. Height and width were significantly correlated, such that taller specimens were wider (p = 0.004, r^2^ = 0.62, N = 11) (Table 2). Basal diameter significantly correlated with height (p = 0.0003, r^2^ = 0.79, N = 11) and width (p<0.0001, r^2^ = 0.85, N = 11) (Tables 1 and 2). Height correlated with the distance to first branch (p = 0.03, r^2^ = 0.41, N = 11) (Table 2). The weight of the specimen significantly correlated with age, height, weight, and axial and radial growth rates (Table 2).

**Table 2:** Statistics comparing morphological data, ages, and growth rates of *Primnoa pacifica*.

	Age estimate (years)	Height (cm)	Width (cm)	BaseD (mm)	DistToBr (cm)	Wet weight (kg) (N = 9)	Radial growth rate (mm yr^-1^)	Axial growth rate (cm yr^-1^)
**Age estimate (years)**		0.0001*	0.0028*	0.0000*	0.3475	**0.0000**	0.0748	**0.0008**
		**0.83**	**0.65**	**0.90**	0.10	**0.97**	0.31	**0.73**
**Height (cm)**			**0.0041**	**0.0003**	**0.0333**	**0.0100**	0.3930	0.0866
			**0.62**	**0.79**	**0.41**	**0.64**	0.08	0.29
**Width (cm)**				**0.0001**	0.2020	**0.0021**	0.8160	0.0631
				**0.85**	0.17	**0.76**	0.01	0.33
**BaseD (cm)**					0.2821	**0.0003**	0.6523	**0.0089**
					0.13	**0.86**	0.02	**0.55**
**DistToBr (cm)**						0.5543	0.7496	0.8717
						0.05	0.01	0.00
**Wet weight (kg) (N = 9)**							**0.0296**	**0.0022**
							**0.51**	**0.76**
**Radial growth rates (mm yr**^**-1**^**)**								0.0689
								0.32
**Axial growth rates (cm yr**^**-1**^**)**								

Age estimates for all specimens are derived from visual counts of annual growth bands. Reported is the p-value and r^2^ for each comparison. All statistics are based on a linear model, except those indicated with an asterisk, which are based on a logarithmic model. N = 11 unless otherwise noted. Significant relationships are in bold.

**Fig 3:**
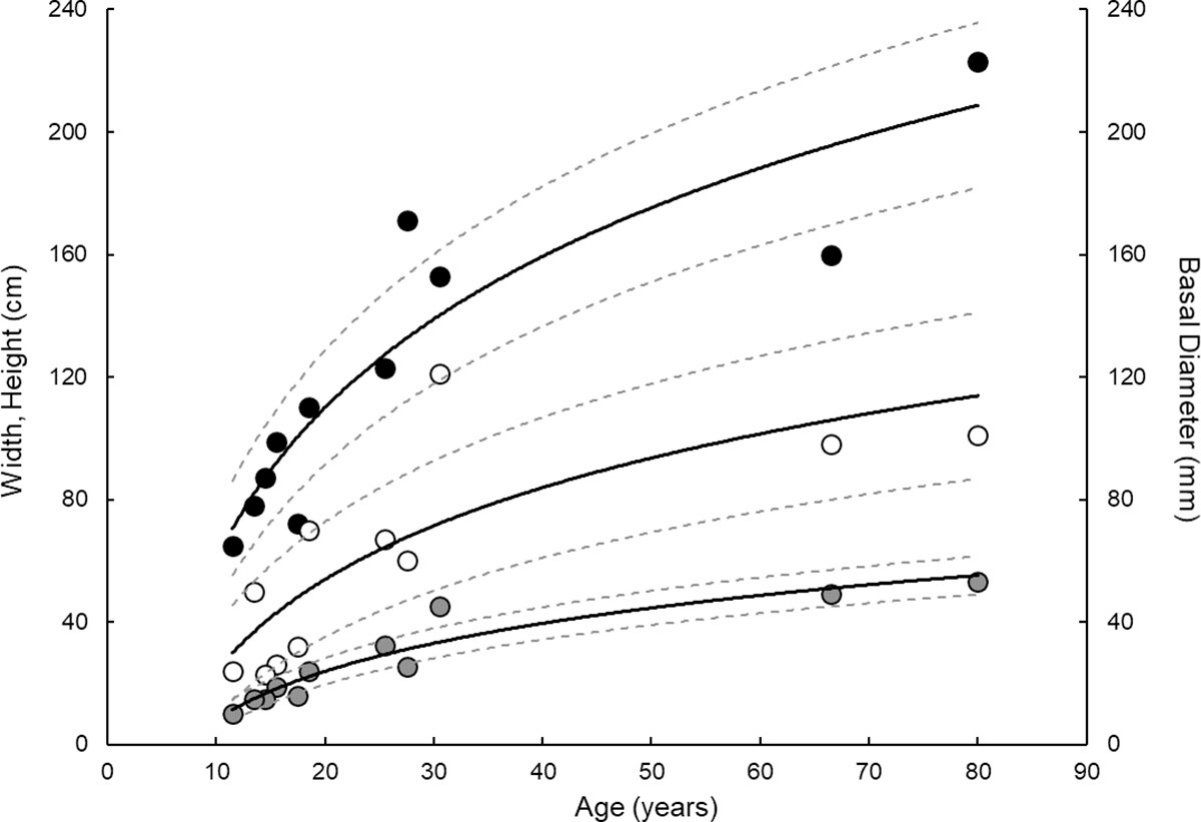
Height (black circles), width (white circles), and basal diameter (grey circles) of *Primnoa pacifica* decrease logarithmically with age. Statistics are presented in Table 2, Eqs (4)–(6). Dashed lines represent 95% confidence intervals.

Age estimates based on annual band counts ranged from 12 ± 2 to 80 ± 1 years. These age estimates correlated with all of the morphological data, except distance to first branch (Table 2). Using a logarithmic model, older specimens were taller (p<0.0001, r^2^ = 0.83, N = 11), wider (p = 0.0028, r^2^ = 0.65, N = 11), and had a larger basal diameter (p<0.0001, r^2^ = 0.90, N = 11) (Table 2; Fig 3). A logarithmic model provided the best fit between age and morphological data, excluding weight (Fig 3). Thus height, width, and basal diameter can be used to determine specimen age in years (± standard error) using the following equations:

$$Age\;(\pm 21)=e^{\frac{Height+109.8}{72.6}}$$ (4)

$$Age\;(\pm 21)=e^{\frac{Width+79.4}{44.1}}$$ (5)

$$Age\;(\pm 5)=e^{\frac{Basal\;Diameter+45.8}{23.0}}$$ (6)

Axial growth rates varied from 2.41 to 6.39 cm year^-1^ and radial growth rates varied from 0.33 to 0.74 mm year^-1^ (Table 3). Using a linear model, axial growth rates significantly inversely correlated with age (p<0.001, r^2^ = 0.73, N = 11) and basal diameter (p = 0.0089, r^2^ = 0.55, N = 11), and positively correlated with specimen weight (p = 0.0022, r^2^ = 0.76, N = 9) (Table 2). Radial growth rates only significantly varied with specimen weight (p = 0.030, r^2^ = 0.51, N = 9) (Table 2).

**Table 3:** Reported ages, radial and axial growth rates of *Primnoa pacifica*. Radiometric age estimates for Andrews et al. (2002) [26] and Williams et al. (2007) [35] were derived from ^210^Pb. Radiometric age estimates for Aranha et al. (2014) [34] were derived from ^14^C. OCNMS is the Olympic Coast National Marine Sanctuary. Shiribeshi seamount is located in the Sea of Japan off the western coast of Hokkaido, Japan. Annual bands were ambiguous in specimens missing age estimates from growth bands (column 3).

Location	Colony name	Age estimate (annual bands)			Age estimate (radiometric)			Radius (mm)	Radial growth rate (mm year^-1^)			Height (cm)	Axial growth rate (cm year^-1^)			Reference
Fairweather Ground	WPA 001	26	±	2	----			16.3	0.64	±	0.05	123	4.82	±	0.37	This study
Fairweather Ground	WPA 002*	28	±	2	42	±	0.1	12.8	0.46	±	0.04	171	6.22	±	0.44	This study
Fairweather Ground	WPA 003	19	±	2	----			12.0	0.65	±	0.07	110	5.95	±	0.62	This study
Fairweather Ground	WPA 004	80	±	1	66	±	1	26.5	0.33	±	0.00	223	2.79	±	0.03	This study
Dixon Entrance	WPB 005	67	±	4	38	±	1	24.5	0.37	±	0.02	160	2.41	±	0.14	This study
Dixon Entrance	WPB 006	16	±	2	----			9.5	0.61	±	0.08	99	6.39	±	0.79	This study
Dixon Entrance	WPB 007	15	±	1	----			7.5	0.52	±	0.04	87	6.00	±	0.39	This study
Portlock Bank	GOA 004	14	±	2	----			7.5	0.56	±	0.08	78	5.78	±	0.81	This study
Shutter Ridge	GOA 011	12	±	2	----			5.0	0.43	±	0.08	65	5.65	±	0.93	This study
Shutter Ridge	GOA 022	18	±	1	----			8.0	0.46	±	0.03	72	4.11	±	0.22	This study
Shutter Ridge	GOA 067	31	±	2	----			22.5	0.74	±	0.05	153	5.02	±	0.32	This study
Dixon Entrance	R1153-0003	46	±	4	46	±	26	10.00	0.22	±	0.08	----	----			Aranha et al., 2014
Dixon Entrance	R1155-0012	34	±	2	34	±	11	19.50	0.57	±	0.13	----	----			Aranha et al., 2014
Dixon Entrance	R1155-0013	30	±	3	119	±	47	9.00	0.30	±	0.07	----	----			Aranha et al., 2014
Dixon Entrance	R1156-0004	22	±	2	22	±	6	5.00	0.23	±	0.05	----	----			Aranha et al., 2014
Dixon Entrance	R1156-0016	16	±	1	49	±	13	4.60	0.29	±	0.08	----	----			Aranha et al., 2014
OCNMS	R1162-0015	45	±	2	75	±	15	18.75	0.42	±	0.06	----	----			Aranha et al., 2014
OCNMS	R1162-0016	31	±	2	51	±	11	16.50	0.53	±	0.06	----	----			Aranha et al., 2014
OCNMS	R1162-0005	40	±	2	62	±	10	13.00	0.33	±	0.03	----	----			Aranha et al., 2014
OCNMS	R1165-0002	21	±	4	87	±	4	8.10	0.39	±	0.08	----	----			Aranha et al., 2014
Dixon Entrance	Colony 1	##			112			----	0.18	±	0.002	----	1.74	±	0.19	Andrews et al., 2002
Dixon Entrance	Colony 2	----			----			----	----			----	2.32	±	0.09	Andrews et al., 2002
Portlock Bank, Alaska	PAL	124	±	3	195	±	5	17	0.14			----	----			Williams et al., 2007
Southeast Alaska	P88	----			123	±	5	23	0.19			----	----			Williams et al., 2007
Eastern Aleutian Islands	P26	----			43	±	0.6	16	0.37			----	----			Williams et al., 2007
Shiribeshi Seamount	1	5	±	3	----			0.7	0.13			----	----			Matsumoto, 2007
Shiribeshi Seamount	2	8	±	2	----			0.9	0.11			----	----			Matsumoto, 2007
Shiribeshi Seamount	3	5	±	2	----			1.0	0.19			----	----			Matsumoto, 2007
Shiribeshi Seamount	4	9	±	2	----			1.2	0.13			----	----			Matsumoto, 2007
Shiribeshi Seamount	5	12	±	2	----			1.3	0.11			----	----			Matsumoto, 2007
Shiribeshi Seamount	6	14	±	3	----			1.7	0.12			----	----			Matsumoto, 2007
Shiribeshi Seamount	7	15	±	2	----			2.0	0.14			----	----			Matsumoto, 2007
Shiribeshi Seamount	8	18	±	6	----			2.2	0.12			----	----			Matsumoto, 2007
Shiribeshi Seamount	9	29	±	2	----			2.8	0.10			----	----			Matsumoto, 2007
Shiribeshi Seamount	10	40	±	2	----			5.4	0.13			----	----			Matsumoto, 2007

*^14^C results for WPA 002 could also yield an age of 49.1 years with radial growth rates of 0.31 mm yr^-1^ and axial growth rate of 4.12 cm yr^-1^.

### Band count comparisons

In four specimens ranging in age from 12 to 80 years, based on annual band counts, the number of sub-annual dissected bands ranged from 290 ± 28 to 1589 ± 124, while the number of sub-annual photographed bands ranged from 152 ± 10 to 1131 ± 45 (Table 4). The number of sub-annual photographed growth bands positively correlated with the number of annual growth bands, such that there is an average of 14 ± 0.8 sub-annual bands for every annual band (p = 0.0002, r^2^ = 1.0, N = 4). Conversely, there is an average of 18 ± 5. Sub-annual dissected growth bands for every annual growth band, although this relationship was not statistically significant (p = 0.096, r^2^ = 0.82, N = 4). The number of dissected band counts increased linearly with sub-annual photographed bands and in most cases was greater than the number of sub-annual photographed band counts, although this relationship was also not significant (p = 0.103, r^2^ = 0.81, N = 4) (Table 4).

**Table 4:** Annual, sub-annual dissected, and sub-annual photographed band counts for four *Primnoa pacifica* colonies in this study.

Sample ID	Annual Band Count			Sub-annual Dissected Band Count			Sub-annual Photographed Band Count		
WPA 002	28	±	2	477	±	64	399	±	36
WPA 004	80	±	1	1589	±	124	1131	±	45
WPB 005	67	±	4	790	±	93	960	±	72
GOA 011	12	±	2	290	±	28	152	±	10

### Radiometric age determination

Measured Δ^14^C values ranged from -93.7 ± 1.3 to 89.4 ± 2.0 (Table 5) and were consistent with previously published regional Δ^14^C records from the North Pacific Ocean [23, 37] (S1 Fig in S1 File). The ages of the corals determined from the timing of the bomb-curve radiocarbon ranged in age from 38 ± 1 (WPB 005) to 66 ± 1 (WPA 004) years old. Thus, based on ages and collection year, the corals started growing between 1949 and 1977.

**Table 5:** Δ^14^C and ^210^Pb_ex_ values for radiocarbon and lead-210 dating, respectively, and resulting ages from each dating method for specimens WPA 002, WPA 004, and WPB 005. The banding frequency and growth rate for each specimen was calculated from the radiocarbon ages. The brackets around the ^210^Pb_ex_ values represent the possible range of bands associated with this measurement.

Sample ID & sub-section	Sub-annual dissected band count	Δ^14^C (‰)	Age from radiocarbon (years)	Frequency (growth band year^-1^)	Radial growth rate (mm yr^-1^)	Vertical growth rate (cm yr^-1^)	^210^Pb_ex_ (dpm/g)	Age from ^210^Pb (years)
**WPA 002**								
(Outer) 1	6.5 ± 2	-5.8 ± 2.0	0	0	---	---	2.52 ± 0.96	---
2	241 ± 56	31.5 ± 1.5	38.2 ± 0.6	6.1	---	---	5.46 ± 1.28	---
(Center) 3	477 ± 64	43.6 ± 1.7	41.5 ± 0.1	16.1	0.26	3.49	2.82 ± 1.38	---
(Center)* 3	477 ± 64	43.6 ± 1.7	49.1 ± 0.4	53.6	0.307	4.12		
**WPA 004**								
(Outer) 1	13.5 ± 2	-12.2 ± 1.8	0	0	---	---	1.51 ± 0.73	0
2	534 ± 38	68.1 ± 1.6	50.0 ± 0.3	11.6	---	---		
3	712 ± 47	80.0 ± 2.2	47.7 ± 0.3	81.5	---	---		
4	869 ± 19	89.4 ± 2.0	47.0	203.5	---	---	0.90 ± 1.76	16.5
5	1070 ± 81	75.3 ± 1.8	48.1 ± 0.1	177.1	---	---		
6	1210 ± 6	-22.5 ± 1.9	51.7 ± 1.0	39.5	---	---	0.24 ± 0.69	59.7
7	1332 ± 33	-56.1 ± 1.8	53.8 ± 0.2	56.4	---	---		
(Center) 8	1589 ± 124	-93.7 ± 1.3	65.7 ± 1.0	17.5	0.404	3.4		
**WPB 005**								
(Outer) 1	23 ± 10	5.2 ± 1.8	0 ± 0.7	0	---	---	3.57 ± 0.96	0
2	265 ± 52	87.2 ± 1.6	19.5	12.4	---	---		
3	348 ± 11	27.5 ± 1.9	22.4 ± 0.1	29.3	---	---	3.54 ± 1.28	0.3
4	416 ± 28	-58.3 ± 1.7	26.6 ± 0.2	16.1	---	---		
5	534 ± 106	-91.0 ± 1.4	35.4 ± 1.4	13.3	---	---	1.58 ± 1.23	26.2
(Center) 6	790 ± 93	-93.5 ± 1.6	38.1 ± 1.3	83.6	0.644	4.2		

*WPA 002 center Δ^14^C value yielded two ages depending if the sample was placed on the rise or the decline of the bomb carbon.

The radiocarbon-derived coral ages were similar to, but consistently older, than the ages derived from the lead-210 dating (Table 5). WPA 002 has two data points for the oldest value (S1 Fig in S1 File) because the innermost Δ^14^C value in this specimen could reflect either rising or declining bomb Δ^14^C values. Based on growth rates and comparisons with the other corals, we estimate that the older value is more likely accurate and estimate the ^14^C-derived coral age as 42 ± 0.1 years old.

^210^Pb_ex_ values ranged from 0.24 ± 0.69 to 3.57 ± 0.96 dpm/g (desentigrations per minute / gram) and decreased as expected from the outside of the colony toward the inside for corals WPA 004 and WPB 005 (Table 5; S2 Fig in S1 File). Eq (2) calculated the age of WPA 004 to between 28.4 and 59.7 and WPB 005 to be 26.2 (+ 38.4/– 10.9) years old. ^210^Pb dating for sample WPA 002 was inconclusive because ^210^Pb_ex_ activity increased from the outside of the coral toward the inside (Table 5; S2 Fig in S1 File).

### Age estimation validation

**Fig 4:**
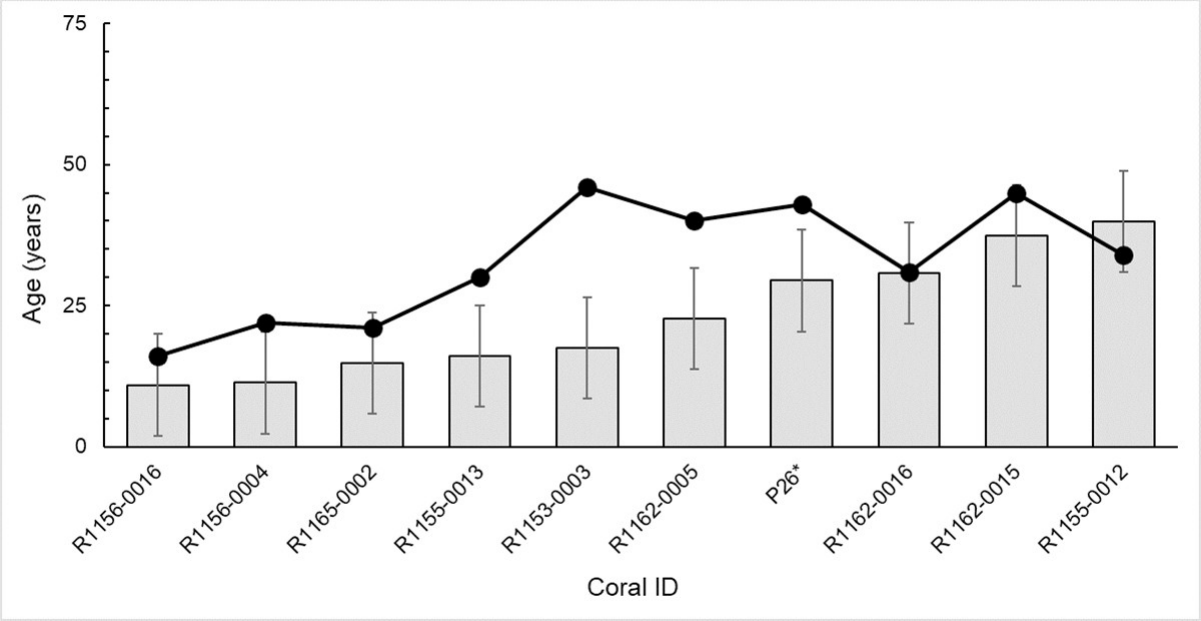
Age estimations based on basal diameter (light grey bar) compared to age (annual bands; black line) of *Primnoa pacifica* <100 years old from previous studies (Aranha et. al. 2014 [34]; Williams et al. 2007 [35]). Age estimations were calculated using Eq 6 which translates basal diameter into a calculated age. Error bars show standard deviation. *Age based on lead-210 dating not annual growth bands.

In previously collected and aged corals (Table 2; corals from Aranha et al., 2104 [34] and Williams et al., 2007 [35]), we find that the calculated ages using Eq (6) were similar in magnitude but underestimated age (on average ~ 10 years) relative to the reported ages (Fig 4).

**Fig 5:**
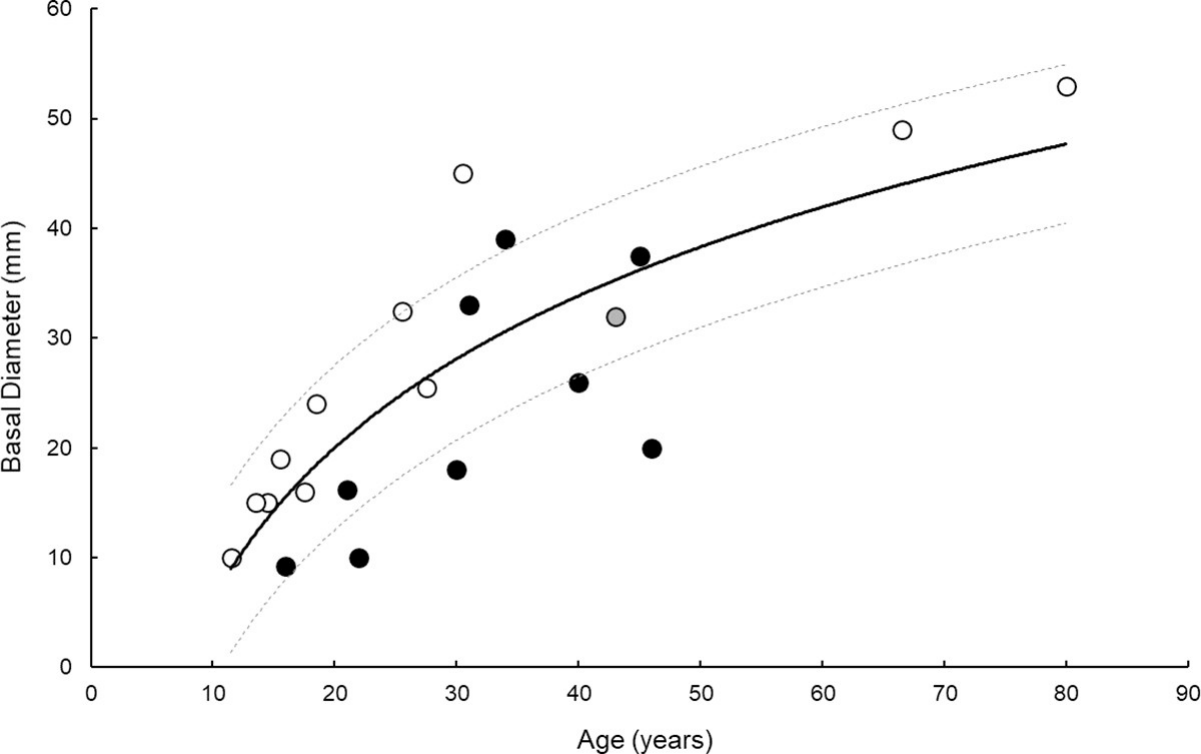
Basal diameter of *Primnoa pacifica* corals from this study (white circles), Aranha et al., 2014 [34] (black circles), and Williams et al., 2007 [35] (grey circles) decrease logarithmically with age (Eq (7), r^2^ = 0.66, p<0.001, N = 21). Corals over a century in age were excluded from this analysis.

**Fig 6:**
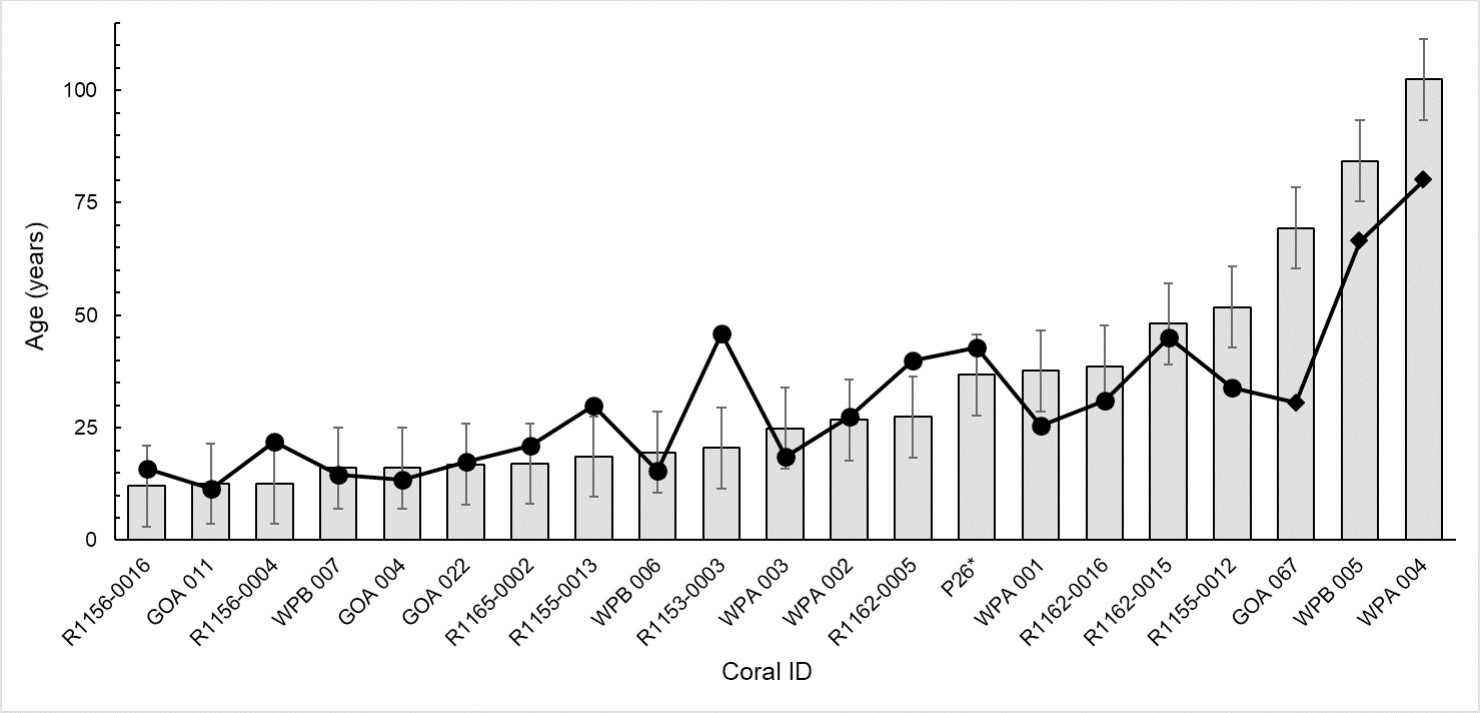
Age estimations based on basal diameter (grey bar) compared to age (annual growth bands; black line) for 21 *Primnoa pacifica* colonies <100 years old from three studies (This study; Aranha et al. 2014 [34]; Williams et al. 2007[35]). Age estimations were calculated using Eq (7). Colonies are ordered from left to right based on increasing basal diameter measurements; diamond markers indicate a basal diameter of 39 mm or more. *Age based on lead-210 dating not annual growth bands.

Thus, we revised the age estimate equation to include the corals with reported basal diameters from the Aranha et al. (2014) [34] and Williams et al. (2007) [35] for corals < century old:

$$Age\;(\pm 8)=e^{\frac{Basal\;Diameter+41.9}{20.5}}$$ (7)

that is inclusive of corals from the northeast Pacific Ocean (Fig 5; N = 21). Using this new regional equation, the average difference between age estimation from annual band counts or ^210^Pb dating versus basal diameter declined to only three years (Fig 6).

**Fig 7:**
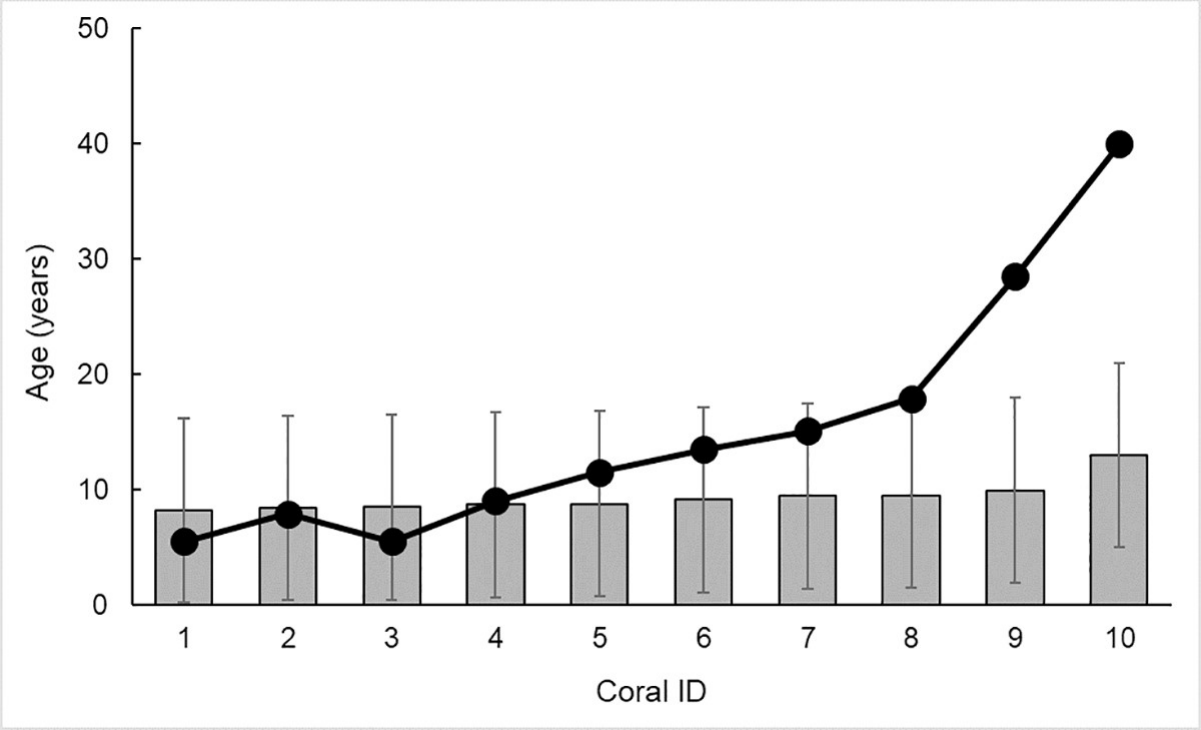
Age estimations based on basal diameter (grey bar) compared to age (annual growth bands; black line) for ten (n = 10) *Primnoa pacifica* colonies collected from the Sea of Japan [39]. Age estimations were calculated using Eq (7). Colonies are ordered from left to right based on increasing basal diameter measurements.

We evaluated Eq (**7**) by calculating age in ten *P*. *pacifica* colonies collected from the Sea of Japan [39]. We find that the age estimated from basal diameter underestimated age compared to the annual band counts by an average of seven years, although this was largely driven by older corals with wider diameters (Fig 7). In coral IDs 1 through 7, the basal diameter underestimated age by three years.

## Discussion

The results of this analysis indicate a strong correlation between age and size (height, width, and basal diameter) of *Primnoa pacifica* (Table 2; Fig 3), and thus provide support for development of non-destructive techniques to estimate the age of younger (< century) specimens in situ. In this species of gorgonian coral, the growth rates declined logarithmically with age such that younger corals grew faster than older corals. This is consistent with other species of gorgonians [32, 40, 41].

Radial growth rates were similar to those reported in previous studies, albeit on the higher end of the reported ranges (Table 3). Axial growth rates were higher than previously reported for two colonies, both of which were older than a century (Table 3). Thus, there is significant variability in radial and axial growth rates, depending the age of the coral. The faster growth rates reported here likely reflect the younger age of the corals. Because growth logarithmically declines with age (Fig 3), eventually reaching a plateau–the age estimate techniques developed here are less useful for older corals and may underestimate the age of very old corals.

### Age estimation validation

Age estimated from basal diameter largely agreed with age from annual band counts in a suite of corals from the northeast Pacific (Fig 6). Discrepancies between the age estimation and annual band counts was the greatest for the oldest specimens with the largest diameters. We hypothesize that smaller corals represent colonies with minimal branching and symmetric radii. In contrast, the larger diameter colonies have more asymmetric basal growth (in which the basal diameter may not reflect 2 x radius) and often with many branches (e.g., specimen WPB 005, Fig 2). Thus Eq 7 is most effective for colonies with basal diameter < 39 mm to provide approximate age estimates for *P*. *pacifica* corals in the North Pacific Ocean.

When the northeast Pacific regional age estimation (Eq 7) was applied to corals from the western Pacific Ocean, basal diameter underestimated age in the oldest colonies. These colonies were, on average, collected from colder, deeper waters (350–505 m; 0.6–0.7°C) and exhibited slower radial growth rates (0.1–0.19 mm year^-1^) than those from the northeast Pacific Ocean [39]. Thus, growth rates may plateau earlier in these corals than in the northeast Pacific Ocean.

Some coral colonies in this study were collected remotely with fishing techniques, which tends to result in fragments, or portions, of a full colony, such that accurate height and width measurements are unavailable, so in-situ age estimations were not possible based on these properties. However, the equations can also be retroactively applied to previous studies that collected morphological data via video analysis but that did not collect corals for age analyses. A Stone (2014) [3] study used video with scaling lasers to categorize red tree corals into four size (height) categories: <0.5 m (small), 0.5–1 m (medium), 1–2 m (large), and >2 m (very large). The height categories can be transformed into the following approximate age ranges using Eq (4): 0–9 years, 9–18 years, 18–71 years, and 71+ years. Equs (4), (5) and (**7**) provide approximate age estimations for corals based solely on morphological data derived from in situ observations. The age estimates are most accurate for the smaller, younger corals (< century) because of the plateau in growth with age. Using this technique, it is possible to identify the locations of gorgonian thickets where recruitment is particularly high (many younger colonies), or colonies may serve as a source of the recruits (many larger colonies), and to locate climax communities where all age classes are well represented.

### Insights into coral dating

Three techniques determined the ages of a subset of the collected corals: annual growth bands were counted in 11 corals, and ^14^C and ^210^Pb dating techniques were performed on three corals. In some cases, results were ambiguous. In *P*. *pacifica*, growth bands form annually but are sometimes difficult to distinguish. Furthermore, radial growth does not always form concentrically, meaning one direction of the coral can form more growth bands than other directions (Fig 2). Thus, most researchers count along the axes of maximum growth yielding the highest number of growth bands. In *P*. *pacifica*, annual growth bands form as clumps of gorgonin bands with some contributions of calcite. Older *P*. *pacifica* corals form significant calcite buildups that mask growth bands; consequently, radiometric estimates of coral age typically exceed those of ages derived from growth bands for older corals [34].

The modern use of radiocarbon dating relies on the identification of the ^14^C bomb curve signature from thermonuclear bomb detonations in the 1950-60s. In the three corals analyzed for ^14^C content, identification of bomb-derived carbon provided age constraints on the timing of skeletal growth. The coral sample sizes analyzed for ^14^C typically included multiple years of growth, smoothing any seasonal variability in ocean ^14^C so that the coral ^14^C values should align with the regional ^14^C bomb curves. However, exact placement on that curve is subject to uncertainty, which is compounded by the analytical uncertainty of the ^14^C measurement. There are two instances in this study where these uncertainties impact age estimates. In WPA 002, the earliest ^14^C value measured from the core could either fall on the rising or declining limb of the bomb carbon curve (S1 Fig in S1 File). We assign it to the rise of the curve because otherwise radial growth rates are unreasonably high for the earliest part of this coral’s growth. In WPB 005, the coral was collected with no living tissue at the base of the specimen; thus, the outer layers of the skeleton were likely not formed immediately prior to collection. The ^14^C value for the outer layers is higher than the outer layers of the live collected WPA 002 and WPA 004, indicating it died some time prior to collection. However, because the slope of the decline of the ^14^C bomb curve flattens toward recent time, the placement of this sample on the curve has large uncertainty with time. Aligning the point exactly on the regional bomb curve gives an age of 38 years for the coral, which yields potentially unrealistically high growth rates. In this colony, annual band counts suggest a much older coral, with a death of only a few years prior to collection. As a result, the strength of ^14^C dating is most evident for specimens with known collection date, and with an age range that encompass the full bomb curve (extending prior to the mid-1950s).

The ^210^Pb dating technique provides only rough age estimates. For example, ^210^Pb of a *P*. *pacifica* colony yielded an age range of 78 to 193 years with 95% confidence [26]. ^210^Pb dating can also yield inconclusive results, potentially in corals that are collected dead, although a mechanism of why is unknown [26]. Here, ^210^Pb yields an age for the dead colony WPB 005, albeit this age may be unrealistically young when viewed in the context of specimen size. In contrast, ^210^Pb yielded inconclusive results for the small live-collected coral WPA 002. Due to the large uncertainty around ^210^Pb, we propose that ^14^C and growth band counting are preferred methods for determining coral age in this species.

### Sub-annual growth bands

We closely examined the sub-annual banding using two techniques: growth bands counted through physical dissection of a cross-section and those visible in photographs of polished cross-sections. The number of dissected growth bands counted was in most cases much greater than the number counted in photographs, although the opposite was true for specimen WPB 004 (Table 4). The skeletal bands were difficult to count, which likely contributed to some of the variation within each counting method–particularly with the photographed bands. Fused growth bands could, in some cases, be carefully peeled apart and counted, although more commonly a peeled fused section included numerous bands that could not be separated. These bands were likely not individually visible in the photographed sections; thus, they were not included in the photographed band counts.

We compared the number of sub-annual bands with the age of each coral to determine banding frequency. Overall, photographed bands average 14 ± 0.80 sub-annual bands year^-1^ and dissected bands average 18 ± 5.2 sub-annual bands year^-1^. We did not count the number of sub-annual bands per year directly because the annual groupings of sub-annual bands were not visible when physically dissecting the cross-sections nor at the lower magnification needed to count the annual bands in the photographs. However, when compared to ages derived from ^14^C-dating, the sub-annual banding frequency ranged from 200 bands year^-1^ to less than 15 bands year^-1^ for specimen WPA 004, 80 bands year^-1^ to less than 15 bands year^-1^ for specimen WPB 005, and over 15 bands year^-1^ to less than 10 bands year^-1^ for specimen WPA 002 (Table 5). Thus, the banding frequency can be highly variable through time. Spring tides (26 per year) have been proposed as a primary driver of sub-annual banding due to the influx of sedimentary organic layer [9]. However, the variability in frequency and the number of bands per year is not consistent with only spring tides. We instead hypothesize that high seasonal and interannual variability of primary productivity [42] and/or energy allocation to reproduction [43, 44] combined with spring tides can influence the variable banding and growth pattern. Additional collections of colonies spanning the size range of the species coupled with time series in situ measurements of primary productivity, specifically flux (POC or particulate organic carbon) delivery to the seafloor, and studies on reproductive seasonality of *P*. *pacifica* in the eastern Gulf of Alaska could elucidate the relationship between these factors and the sub-annual skeletal development.

### Growth rates

Reported radial growth rates of *Primnoa pacifica* range from 0.14 to 0.74 mm year^-1^ (Table 3), with the corals in this study on the higher end of that range 0.33 to 0.74 mm year^-1^, compared with 0.22 to 0.57 mm year^-1^ (Aranha et al., 2014 [34]), 0.36 mm year^-1^ (Andrews et al., 2002 [26]), and 0.14 to 0.37 mm year^-1^ (Williams et al., 2007 [35]). Expanding the age–growth comparisons to include all of these studies shows that radial growth rates are lower in older specimens (linear regression; p = 0.004, r^2^ = 0.35, N = 22). The decrease in radial growth rates overtime is also observed in North Atlantic *Primnoa resedaeformis*, a slower growing congener of *P*. *pacifica* [9, 32, 41]. Additionally, reported radial growth rates of *P*. *resedaeformis* (0.083 to 0.215 mm year^-1^) varied based on colony location; colonies in areas with a stronger tidal current exhibited faster radial growth than colonies in areas of weaker current [32]. There was no statistical difference between location and radial growth rates of the *P*. *pacifica* included in this study (one-way ANOVA; F_4,17_ = 1.15, p>0.05).

Axial growth rates ranged from 2.41 to 6.39 cm year^-1^ (Table 3). This is substantially faster than previously reported axial growth rates of 1.74 and 2.32 cm year^-1^ (Andrews et al., 2002 [26]). This difference is easily explained by the decline in growth rates with age, and that the specimens measured by Andrews et al. (2002) [26] were substantially older than those in the present study (age of 114 years is reported for the specimen growing 1.74 cm year^-1^, with no age for the second colony). Age continues to have a strong, significant explanation of axial growth rates when we add this one specimen with both age and axial growth rates to our age–growth comparisons (linear regression; p<0.0001, r^2^ = 0.80, N = 12). A similar decrease in axial growth rates with age was reported in North Atlantic *P*. *resedaeformis*, where it was proposed that young corals (<30 years) grew four times as fast as older corals (>30 years) [41]. Axial growth rates in these *P*. *resedaeformis* studies ranged from 1.00 to 2.61 cm year^-1^ (coral ages ranged from 18 years to 100 years) and were on the lower end compared to *P*. *pacifica* in this study [32, 41].

### Implications for coral conservation

With the ability to now estimate the age of red tree corals in situ we can readily determine how old corals are using non-invasive video survey techniques coupled with mensuration systems such as lasers or stereo-cameras [4, 6]. Such enhanced surveys could quickly determine the age-class structure and consequently maturity status of coral habitats. This information could be used by coastal managers to identify which aggregations are most vulnerable to disturbance from human activities, and which should be highlighted for protection. If age and growth characteristics are phylogenetically constrained, as has been suggested for some taxa [40], then the techniques developed and insights gained in this study could have broader application in the North Atlantic Ocean where another *Primnoa* species (e. g. *P*. *resedaeformis*; [13, 45]) also forms ecologically important habitats.

## Supporting information

S1 File (PDF)
