# Supplementary material for: Understanding growth and age of red tree corals (Primnoa pacifica) in the North Pacific Ocean
Source: PLoS One. 2020 Dec 1;15(12):e0241692. doi: 10.1371/journal.pone.0241692 (PMC7707513; doi:10.1371/journal.pone.0241692)
Supplement: S1 File — (PDF) [file pone.0241692.s001.pdf]

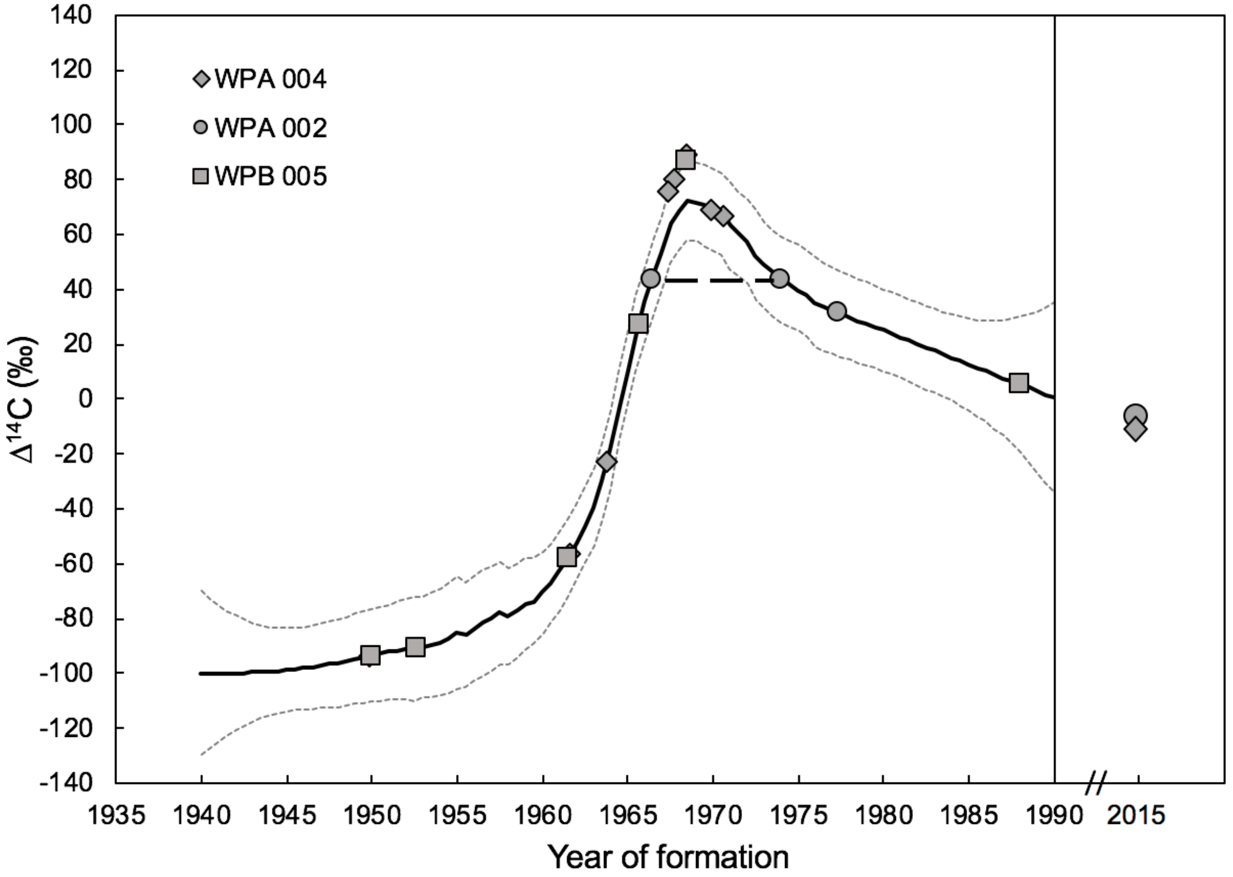

S1 Fig. $\Delta^{14}C$ values associated with calendar year using marine data from Andrews et al. (2013) (solid black line and 95% confidence intervals plotted as grey dashed lines) for samples WPA 002, WPA 004, and WPB 005. The black dashed line indicates that there are two possible years for this $\Delta^{14}C$ value. The 2015 measurements represent the $\Delta^{14}C$ values of the outermost skeletal.

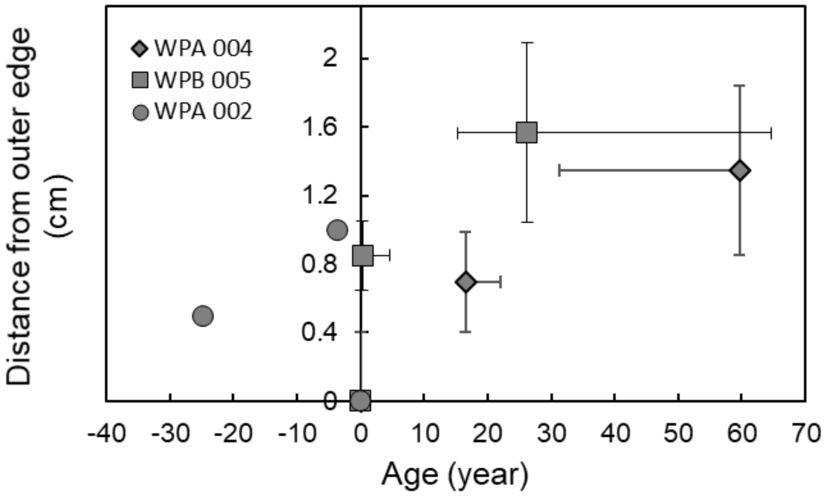

S2 Fig. A) Lead-210 age of *Primnoa pacifica* at three different radial distances for samples WPA 004, WPB 005, and WPA 002. The ages are the average age of the range as indicated by the vertical error bars. Horizontal error bars represent the error associated with $^{210}Pb_{ex}$ values. For WPA 002, $^{210}Pb_{ex}$ values produced negative ages, rendering lead-210 dating for this sample to be inconclusive. Negative ages are calculated when measured ^{226}Ra activity is greater than $^{210}Pb_{ex}$ activity and can arise for several reasons including analytical error from measurements nearing detection limits such as these.
